# Three genes expressed in relation to lipid metabolism considered as potential biomarkers for the diagnosis and treatment of diabetic peripheral neuropathy

**DOI:** 10.1038/s41598-023-35908-9

**Published:** 2023-05-29

**Authors:** Ye Yang, Qin Wang

**Affiliations:** Department of Geriatrics and Cadre Ward, Second Affiliated Hospital of Xinjiang Medical University, Ürümqi, 830063 Xinjiang China

**Keywords:** Endocrine system and metabolic diseases, Metabolic disorders

## Abstract

Diabetic neuropathy is one of the most common chronic complications and is present in approximately 50% of diabetic patients. A bioinformatic approach was used to analyze candidate genes involved in diabetic distal symmetric polyneuropathy and their potential mechanisms. GSE95849 was downloaded from the Gene Expression Omnibus database for differential analysis, together with the identified diabetic peripheral neuropathy-associated genes and the three major metabolism-associated genes in the CTD database to obtain overlapping Differentially Expressed Genes (DEGs). Gene Set Enrichment Analysis and Functional Enrichment Analysis were performed. Protein–Protein Interaction and hub gene networks were constructed using the STRING database and Cytoscape software. The expression levels of target genes were evaluated using GSE24290 samples, followed by Receiver operating characteristic, curve analysis. And Gene Ontology (GO) and Kyoto Encyclopedia of Genes and Genomes (KEGG) enrichment analysis were performed on the target genes. Finally, mRNA-miRNA networks were constructed. A total of 442 co-expressed DEGs were obtained through differential analysis, of which 353 expressed up-regulated genes and 89 expressed down-regulated genes. The up-regulated DEGs were involved in 742 GOs and 10 KEGG enrichment results, mainly associated with lipid metabolism-related pathways, TGF-β receptor signaling pathway, lipid transport, and PPAR signaling pathway. A total of 4 target genes (CREBBP, EP300, ME1, CD36) were identified. Analysis of subject operating characteristic curves indicated that CREBBP (AUC = 1), EP300 (AUC = 0.917), ME1 (AUC = 0.944) and CD36 (AUC = 1) may be candidate serum biomarkers for DPN. Conclusion: Diabetic peripheral neuropathy pathogenesis and progression is caused by multiple pathways, which also provides clinicians with potential therapeutic tools.

## Introduction

Diabetes mellitus (DM) is a chronic metabolic disease, of which the incidence is increasing year on year and has become the seventh leading cause of death worldwide. Over the past 40 years, among all chronic diseases,the prevalence of diabetes has increased most significantly among Chinese adults. Studies have found that the prevalence of diabetes among Chinese adults has increased from 0.67% in 1980 to over 12.8% in 2020^1^.According to the International Diabetes Federation (IDF) diabetes map published in 2019, the total cost of diabetes-related health care in China was US$109 billion, making it the second highest country in the world in terms of diabetes-related health care expenditures^2^.Diabetes has become a global health care issue. According to the latest facts and figures from the International Diabetes Federation, 463 million people worldwide have diabetes and 374 million are at increased risk of developing type 2 diabetes^3^, with up to 20–60% of people with diabetes developing painful diabetic peripheral neuropathy(PDPN)^4^.Painful diabetic peripheral neuropathy (PDPN) is a diabetic peripheral neuropathy characterised by painful symptoms and is highly disabling, leading to reduced physical function, increased financial burden and psychological and social restrictions of the patients. In addition the disease can lead to anxiety, depression and sleep disturbances, which can seriously affect the quality of life of patients.

Of the above complications, diabetic neuropathy is one of the most common chronic complications, with approximately 50% of people with diabetes having diabetic neuropathy^5^. Diabetic neuropathy can take many forms, including sensory, motor, focal/multifocal and autonomic neuropathy^6–8^. The most common type is Diabetic peripheral neuropathy (DPN) which accounts for approximately 75% of diabetic neuropathy^9–13^. DPN is a chronic, symmetrical, progressive disease with early pain, atopic pain and sensory abnormalities. It has a high morbidity and mortality rate and is one of the most significant complications resulting in amputation, disability, death and compromised quality of life^14^.

With recent advances in medical technology, the early detection rate of DPN has been increasing, but there is still some variation in the detection rate of DPN in T2DM patients from one location to another, with a prevalence of 44.58–59.80%.DPN can cause diabetic foot ulcers as the disease progresses, requiring amputation for those with severe ulcers, causing great mental and physical pain as well as a heavy financial burden to the patient ^15^.

Studies have shown that the prevalence of foot ulcers in Chinese diabetics is 4.1%^16^, the amputation rate for foot ulcer patients is 19.03%^17^, and the average length of stay in hospital for diabetic amputees is 11 days longer than for non-diabetic amputees, with medical costs as high as US$1,831^18^.

DPN has distinct symptoms, usually manifesting as distal symmetric sensorimotor polyneuropathy, including neuropathic pain, numbness and burning sensations. In addition, advanced and severe cases may lead to serious consequences, including neurogenic joints, ulcers, fractures, ischaemic gangrene and even death^19–21^ .

Current treatment strategies rely on glucose-lowering drugs and nerve-nourishing medications. However, the therapeutic effect of these drugs on diabetic neuropathy is inadequate, acting primarily through lowering hyperglycaemia rather than through specific and precise targets of diabetic neuropathy. Furthermore, not all patients benefit from these drugs because of the genetic heterogeneity and complexity of the disease. Therefore, the search for new targets to improve the treatment of diabetic neuropathy is an urgent priority.

With the development of bioinformatics, it is increasingly recognized that human diseases are not caused by a single molecular defect but are driven by complex interactions between various molecules. The complexity of these interactions encompasses different types of information, ranging from protein–protein interactions at the cellular molecular level to studies related to gene expression and regulation, metabolic and disease pathways, and drug-disease relationships^22^. Cyber medicine is a rapidly growing and emerging field that combines molecular biology and network science and promises to unravel the causes of human disease and fundamentally change the way humans are treated^23^. Based on network medicine algorithms, protein–protein interactions (PPI)^24,25^ and Weighted Gene Co-expression Network Analysis (WGCNA)^26^ have been successfully applied to the research of pathogenesis of chronic obstructive pulmonary disease^27^, cancer and other diseases^28–31^.

The specific pathogenesis of DPN is still not well understood, but previous studies have shown that disorders of lipid, glucose and protein metabolism in T2DM patients are involved in the disease progression of DPN through different mechanisms that cause microvascular dysfunction and toxic effects on neuronal cells^32,33^. CREBBP, EP300, ME1 and CD36 are also involved in lipid metabolism, but few studies have reported their relevance to the pathogenesis of DPN.

In this study, we aim to explore potential immune-related biomarkers and to elucidate their potential mechanisms, using a bioinformatics approach, to find diabetic neuropathy-related biomarkers that could identify new diagnostic and therapeutic targets for patients with diabetic neuropathy. With GEO public database, we analyzed DEGs in biopsies from DPN patients and healthy controls. Then, to analyze the main biological functions and signalling pathways regulated by DEGs, GO and KEGG analyses were performed to further explore the signalling pathways associated with the disease and explore the molecular mechanisms underlying the occurrence of DPN. Meanwhile, the protein interaction network of co-regulated DEGs was combined with GSEA and ROC analyses to further identify target genes related to DPN diagnosis and treatment, and finally, an mRNA and miRNA correlation network was constructed to screen important miRNAs and gene factors from them.

## Materials and methods

### Data collection and pre-processing

The National Center for Biotechnology Information (NCBI) Gene Expression Omnibus database (https://www.ncbi.nlm.nih.gov/geo/) was used to obtain DPN related gene expression profile data under the keywords "Diabetic neuropathy" or "Diabetic peripheral neuropathy" to obtain a separate gene microarray dataset GSE95849 via the GEOquery package [version 2.54.1]^34^. Downloaded from the GEO database (https://www.ncbi.nlm.nih.gov/U) GSE95849, and based onGPL22448 Phalanx Human lncRNA OneArray v1_mRNA platform, this dataset included tissue samples from six diabetic peripheral neuropathy patients and six healthy participants. The data were normalized for a second time through the limma package of the function. Principal Component Analysis (PCA) was performed on the normalised dataset using R (version 3.6.3). UMAP analysis was performed using the umap package [version 0.2.7.0], and ggplot2 [version 3.3.3] was used to plot Box plot, PCA and UMAP plots, in order to see the clustering between sample subgroups, and finally variance analysis was performed using the limma package^10^ .

### Identification of DEGs associated with diabetic peripheral neuropathy

Samples from GSE95849 were extracted and analysed separately using the limma package [version 3.42.2] to obtain differentially expressed genes (DEGs) between patients with diabetic peripheral neuropathy and healthy participants, and the results were de-duplicated. FDR was used to correct the q-values for multiple hypothesis testing, log2FC|> 1, *p* < 0.05 was statistically significant, and gene IDs were converted to gene symbols according to human genome GRCh38.93. Subsequently, to better understand the DEGs, the differentially expressed genes obtained were applied separately to the R package [version 3.3.3] "ggplot2" for DEmRNA mapping and "ComplexHeatmap [version 2.2.0]" for heat mapping^35^. Finally, the up-regulated DEmRNAs and down-regulated DEmRNAs obtained from the screening were compared with the DEmRNAs identified in the Comparative Toxicogenomics Database, 2021 update (http://ctd.mdibl.org/) database as "Diabetic Neurology"^36^. Diabetic Neuropathy" was used as a keyword to search for genes directly related to diabetic nephropathy. Genes related to amino acid metabolism, glucose metabolism and lipid metabolism were downloaded from the GSEA database^26^ (https://www.gsea-msigdb.org/gsea/msigdb/index.jsp), and overlapping DEGs with consistent up- and down-regulation of expression associated with diabetic neuropathy were taken. Ultimately, VennDiagram [version 3.6.3]", the "ggplot2 package [version 3.3.3]" was used to plot the Venn diagram.

### GO enrichment and KEGG signalling pathway enrichment analysis

The above DEGs were extracted and the DAVID online database (https: //david.ncifcrf.gov/) was used to perform a Gene Ontology (GO) function enrichment analysis with Homo sapiens in the background, providing the required GO function enrichment data. The GO functional enrichment data are annotated and classified according to the functions of the genes: biological process (BP), cellular component (CC), molecular function (MF). At the meantime, the KEGG (Kyoto Encyclopedia of Genes and Genomes) was used for function enrichment analysis of signalling pathway, so as to discover biological pathways that may be involved^37–39^. We set the minimum gene to 10 and the maximum gene to 500, with *p* < 0.05 and FDR < 0.2 considered to be statistically significant, to screen for major enrichment functions and pathways of differential genes^40^. The clusterProfiler package [version 3.14.3] was used for enrichment analysis^41^. The zscore values were calculated using the GOplot package [version 1.0.2], the org.Hs.eg.db package [version 3.10.0] for ID conversion, and the org.Hs.eg.db package [version 3.10.0] for ID conversion^42^ , and finally plotting bubble and circle plots.

### Network analysis of protein–protein interaction (PPI) of common DEGs

We used the STRING database (https://string-db.org/) to present and evaluate PPI networks^43^. The common DEGs screened in this study were imported into STRING, and the STRING analysis tool allowed further exploration of potential associations between these DEGs. The results of interaction node data with joint scores > 0.7 high confidence were imported into Cytoscape (version 3.8.2), and the protein interaction network was analysed for common differentially expressed genes, and visualisation and association analysis^40^. The top 20 genes in the PPI network were then tagged as hub genes using the degree algorithm of the CytoHubba plugin to filter the top 20 genes in key positions in the PPI network^44,45^. Links between all cluster pairs were shown using Spearman correlations, and pairwise correlations between clusters were visualised as chord plots in R (version 3.6.3) using the circlize package [version 0.4.12]^46^. GO and KEGG analyses were also performed on potential hub genes^40,41^.

### Gene set enrichment analysis (GSEA)

To explore biological signalling pathways and key genes, we used the clusterProfiler package [version 3.14.3]^41^ to perform gene set enrichment analysis (GSEA)^47^. The MSigDB Collections gene set database was used as the reference gene set for the species: Homo sapiens, with c2.cp.v7.2.symbols.gmt [Curated]. The corrected normalized enrichment score |NES|> 1, False discovery rate (FDR) < 0.25 and p.adjusted < 0.05 conditions were considered significantly enriched, and relevant enrichment pathways and core genes that play a key role in these enrichment pathways were selected out.

### Intergroup differential expression of diabetic peripheral neuropathy hub genes

Statistical analyses of potential pivotal genes were performed using the R package (version 3.6.3), and differences between DPN and normal participant groups were determined using the student t-test and Weltch's t' test. Data were tested for normality and chi-squared, and t-tests were used if the distribution was close to normal (*p* > 0.05). If the variance of the observed variables in the two groups was equal (*p* > 0.05), the independent samples t-test is used, and if the variance of the observed variables in the two groups was not statistically equal (*p* < 0.05), the Weltch t' test is applied. Finally, a combined plot of point, box and violin plots was visualised using ggplot2 [version 3.3.3], with the significance mark: ns, *p* ≥ 0.05; *, *p* < 0.05; **, *p* < 0.01; ***, *p* < 0.001.

### Diagnostic validation of target genes

In the gene sets GSE95849 and GSE24290, ROC curve analysis was performed using the pROC package to determine the sensitivity and specificity of each of the 16 target genes, the ROC-related information and data for the predictor variables at their respective cut-off values, and to assess the accuracy of the gene for the diagnosis of DPN. The results were quantified with the area under the ROC curve (AUC) and genes with an AUC > of 0.6 were selected as diagnostic genes, again visualised using ggplot2.

### Construction of mRNA-miRNA regulatory networks and prediction of key miRNA

The miRWalk database^48^ (http://mirwalk.umm.uni-heidelberg.de/) Jan/2021 -new update was used for interactions between differentially expressed mRNAs and miRNAs. Based on this, prediction of miRNAs was performed on the miRTarBase database and miRDB database^46^. Candidate miRNAs were obtained from the intersection of the 3 databases^47,48^and Cytoscape 3.8.2 was used to visualize the regulatory network from which the important miRNAs and mRNAs were screened out.

## Results

### Screening for pivotal genes in diabetic peripheral neuropathy

**Figure 1:**
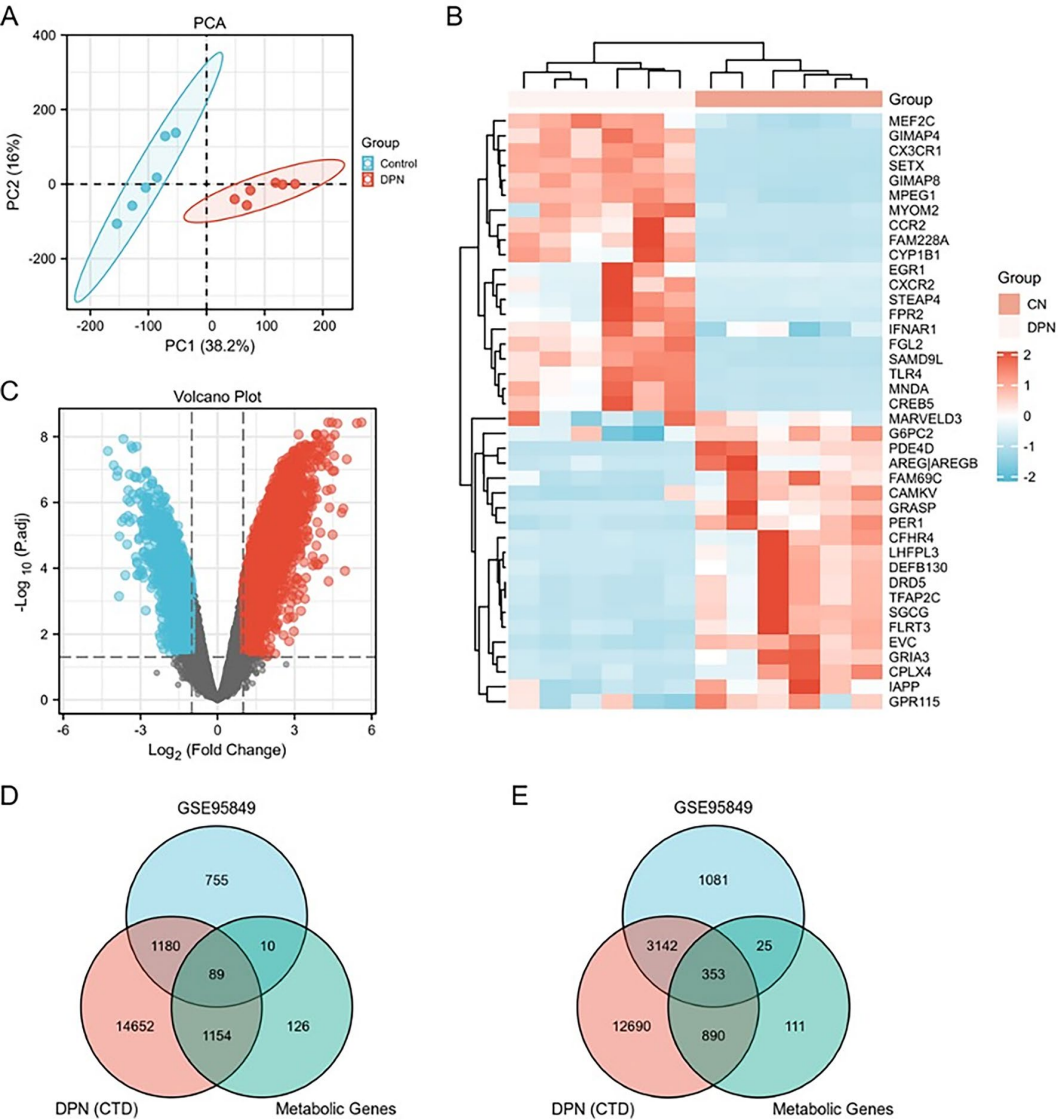
Associated genes and Venn diagrams differentially expressed in DPN and healthy samples. (**A**) Principal component analysis of GSE95849. (**B**) Hierarchical clustering tree heat map of the top 20 up-regulated (red dots) and top 20 down-regulated (blue dots) differentially expressed genes. (**C**) Volcano plot of differentially expressed mRNAs, |logFC|> 1, adj:*p* < 0.05. Up-regulated genes are shown in red, down-regulated genes are shown in blue, and genes with no significant difference are shown in grey. (**D**) Venn diagram showing the number of down-regulated differential genes co-overlapping with DPN-related genes in the CTD database and metabolism-related genes in the GSEA database. (**E**) Venn diagram showing the number of up-regulated differential genes co-overlapping with DPN-related in the CTD database and metabolism-related genes in the GSEA database.

In screening the DEGs, a total of 6 DPN samples and 6 control samples were included in the GEO dataset GSE95849, and this dataset was normalised. A principal component analysis (PCA) of GSE95849 was conducted to demonstrate clustering using scatter plots. Each point in the scatter plot represents one sample each, with 38.2% for PC1 and 16% for PC2, and the plot shows significant differences between the groupings (Fig. 1A). The volcano plot shows that a total of 16,405 differentially expressed genes were identified, of which 9822 were up-regulated and 6583 were down-regulated (Fig. 1C). The heat map shows the top 20 up-regulated genes and the top 20 down-regulated genes (Fig. 1B). Finally, differential analysis were performed by DESeq2, which determined the log2 fold changes, Wald test *p*-values and adjusted *p*-value (FDR) by the Benjamini–Hochberg procedure. Significantly regulated genes were defined as log2FC > 1 or < -1, and FDR < 0.5. According to the screening condition |log2FC|> 1, *p* < 0.05, 4601 genes with up-regulated differences in expression and 2034 genes with down-regulated differences in expression were screened from the GSE9589 dataset. The above differential genes were screened together with 23,971 genes related to diabetic neuropathy from the CTD database and 1404 key genes of glucose metabolism, lipid metabolism and amino acid metabolism from the GSEA database to create a Venn diagram. The results showed that the expression of 442 overlapping DEGs was generally different, with 353 genes being up-regulated and 89 genes being down-regulated (Fig. 1D, E). Because the screened DEGs would contain genes that were inconsistently up- and down-regulated, direct bioinformatic analysis of genes and pathways associated with diabetic peripheral neuropathy disease would confound the effect of false-positive co-expressed genes. To exclude this confounding factor, and to screen for genes that can be used as predictive targets for clinical diagnosis and prognosis, compared with those of normal healthy individuals, up-regulated genes are more clinically feasible to apply and study. Moreover, up-regulated genes are more practical from a diagnostic or therapeutic view point, we thereafter focused our attention on the up-regulated genes.We therefore selected only genes with up-regulated expression among the co-differential genes for analysis.

### Results of GO and KEGG enrichment analysis

**Figure 2:**
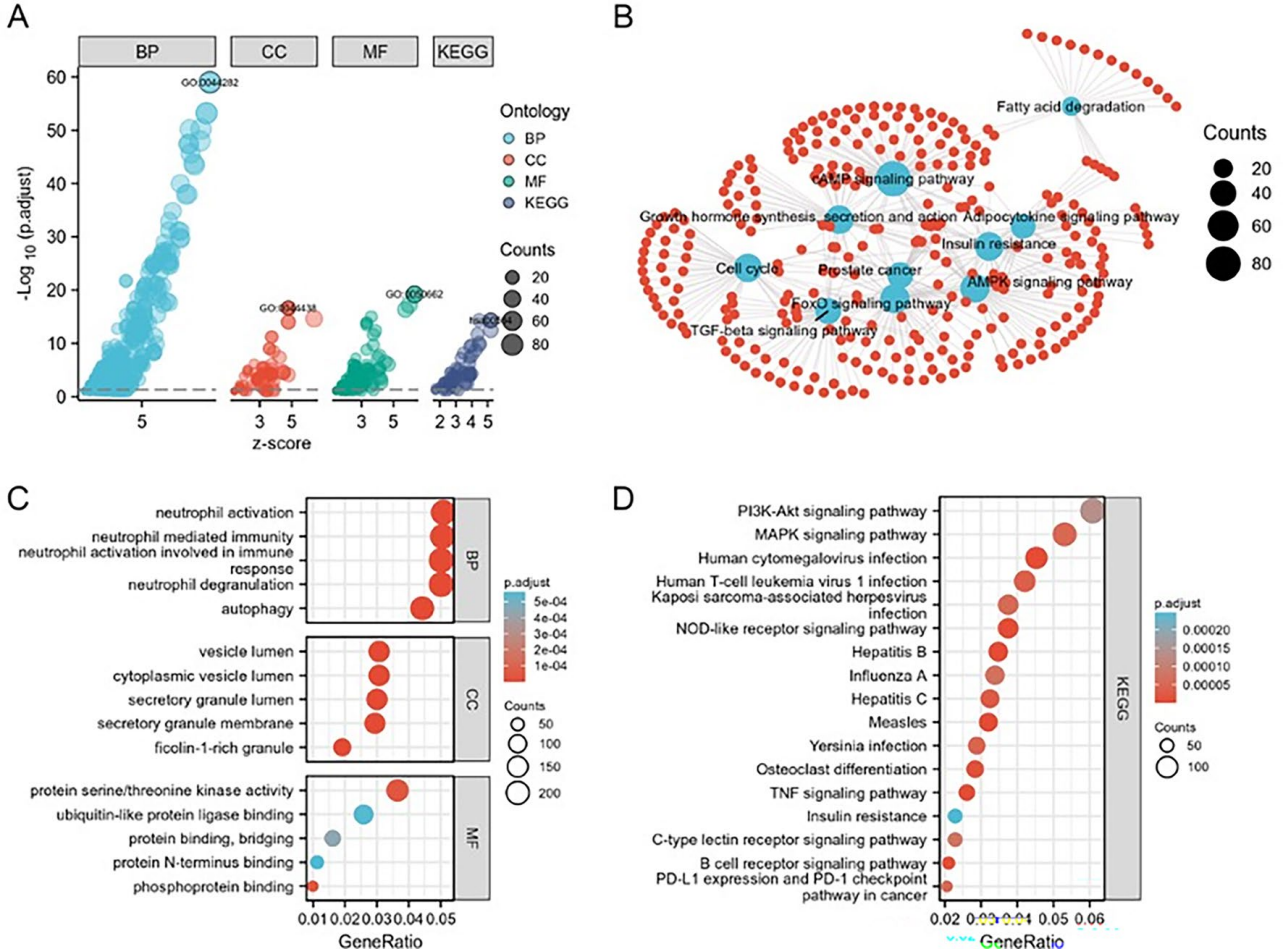
Functional enrichment analysis of 353 up-regulated co-expressed differential genes. (**A**) GO and KEGG enrichment analysis, GO analysis including BP (biological process), CC (cellular component), MF (molecular function). (**B**) Enrichment analysis of the 10 KEGG star pathways. (**C**) Enrichment results for the first 5 GOs, with the X horizontal axis indicating the proportion of DEGs enriched in the GO term. The redder the colour, the larger the corrected *p*-value, and the size of the dot represents the number of enriched genes (**D**). Enrichment analysis of the first 20 KEGG pathways.

In DAVID-based GO biological process and KEGG signalling pathway enrichment annotation analysis on 353 overlapping gene DEGs, meeting the conditions of p.adj < 0.05 & qvalue < 0.2 for significant enrichment, there were 563 BP, 59 CC, 120 MF and 61 KEGG. After arranging them according to the FDR values from smallest to largest, they were visualized as in (Fig. 2A). These DEGs were significantly enriched in small molecule catabolic process, carboxylic acid and organic acid biosynthetic and catabolic process, glycerolipid metabolic and biosynthetic process, phospholipid biosynthetic process, phospholipid metabolic process, glycerophospholipid metabolic process, fatty acid metabolic process, coenzyme metabolic process and other processes(Fig. 2C). In the KEGG enrichment analysis, DEGs were involved in insulin resistance, MAPK signaling pathway, cAMP signaling pathway, FoxO signaling pathway, TNF-beta signaling pathway, Adipocytokine signaling pathway, Growth hormone synthesis, secretion and action, Cell cycle, Glycerophospholipid metabolism, Valine, Leucine and Isoleucine degradation (Val) , Carbon metabolism, Fatty acid metabolism, PPAR signaling pathway, Sphingolipid metabolism, Peroxisome metabolism Peroxisome, Fatty acid degradation, Glycolysis / Gluconeogenesis, Glucagon signaling pathway and other metabolic processes (Fig. 2B,D).

### Construction of PPI networks and identification of hub genes

**Figure 3:**
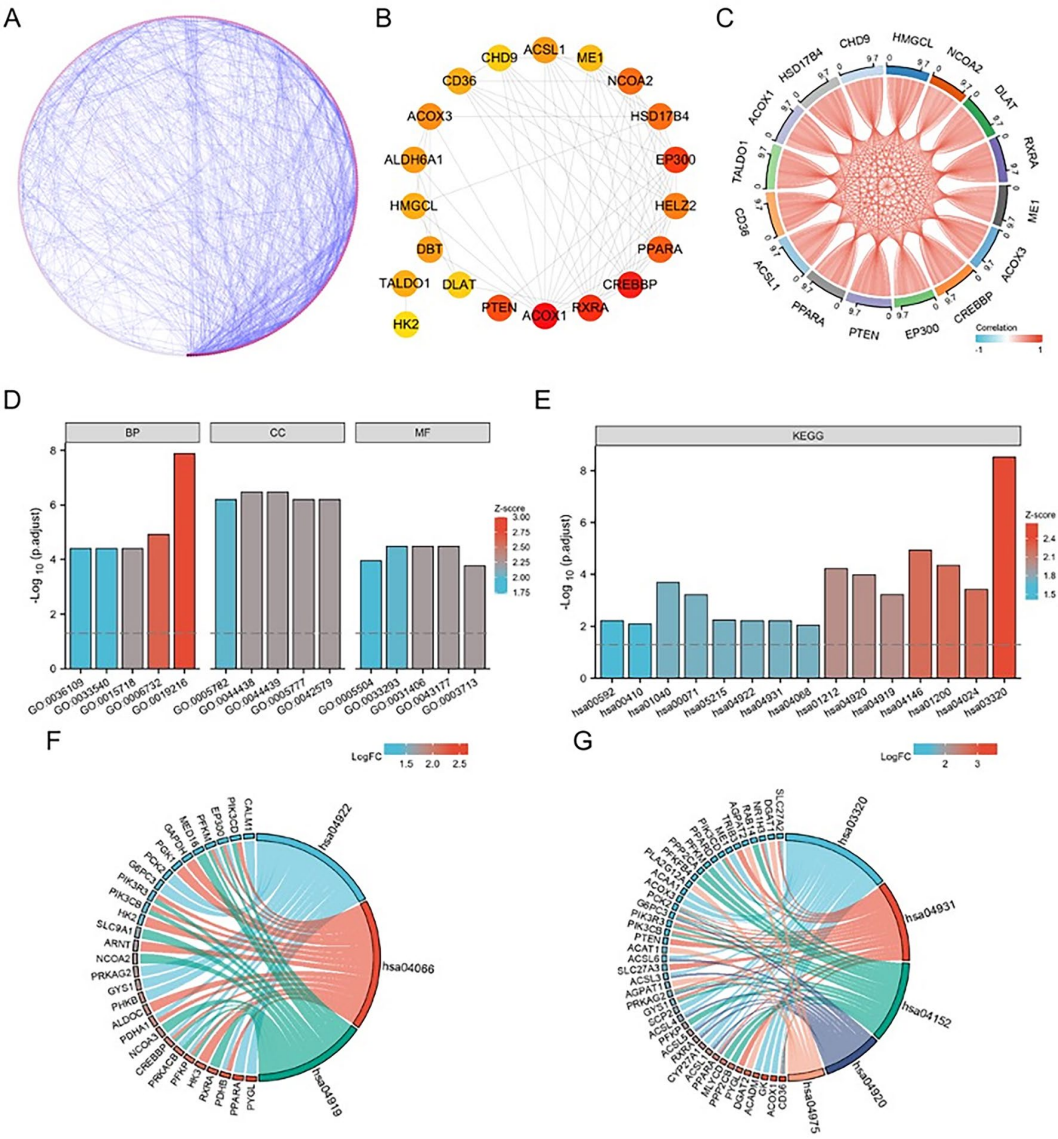
PPI network and associated enrichment analysis. (**A**) PPI network of up-regulated co-expressed genes constructed based on Cytoscape. Nodes represent proteins and edges represent protein interactions. The colour depth of nodes is the degree-value and the colour depth of edges is the combined-score value, both indicating their importance in the network. (**B**) Top 20 Hub genes identified. (**C**) 16 Hub chord diagrams. (**D**) Results of the first 5 GO functional enrichment analyses, including: BP, CC and MF.(**E**) Results of the first 15 KEGG enrichments. KEGG enrichment analysis of 3 hub genes. (**F**) KEGG enrichment results for CREBBP (CBP) and EP300 (P300). (**G**) KEGG enrichment results for CD36.

To understand the interactions between upregulated DEGs, a PPI network was constructed using STRING for co-expressing DEGs (Fig. 3A), and the results were then imported into Cytoscape v.3.8.2 software, and the genes in this network were ranked according to their degree values using the cytoHubba plugin to identify the top 20 hub genes with the highest degree.

With an App called cytoHubba in Cytoscape software (version 3.8.2), we calculated the connectivity degree of each gene and selected the top 20 most central genes in the PPI network。These hub genes were ACOX1, RXRA, CREBBP, PPARA, EP300, HELZ2, NCOA2, ME1, HSD17B4, ACSL1, CHD9, CD36, ACOX3, ALDH6A1, HMGCL, DLAT, DBT, PGM1, PTEN, TALDO1 (Fig. 3B). Correlation analysis was also performed for 20 pivotal genes, and the chord plot showed that 16 of them were positively correlated (Fig. 3C). Subsequently, GO and KEGG enrichment analyses were performed on the 20 pivotal genes. The results showed that most of the 20 pivotal genes were enriched in the regulatory processes of lipid metabolism, amino acid metabolism, PPAR signalling pathway, cAMP signalling pathway, fatty acid metabolism and glucagon signalling pathway related to the development and progression of diabetic peripheral neuropathy, as shown in Figure (Fig. 3 D, E, Tables 1 and 2).

**Table 1:** Results of GO enrichment analysis.

Ontology	ID	Description	GeneRatio	*p*.adjust	*p*.value
BP	GO:0,036,109	Alpha-linolenic acid metabolic process	3/16	3.98e − 05	2.11e − 05
BP	GO:0,033,540	Fatty acid beta-oxidation using acyl-CoA oxidase	3/16	3.98e − 05	2.11e − 05
BP	GO:0,015,718	Monocarboxylic acid transport	5/16	3.98e − 05	2.11e − 05
BP	GO:0,006,732	Coenzyme metabolic process	7/16	1.19e − 05	6.33e − 06
BP	GO:0,019,216	Regulation of lipid metabolic process	9/16	1.29e − 08	6.87e − 09
CC	GO:0,005,782	Peroxisomal matrix	4/16	6.24e − 07	3.59e − 07
CC	GO:0,044,438	Microbody part	5/16	3.36e − 07	1.93e − 07
CC	GO:0,044,439	Peroxisomal part	5/16	3.36e − 07	1.93e − 07
CC	GO:0,005,777	Peroxisome	5/16	6.24e − 07	3.59e − 07
CC	GO:0,042,579	Microbody	5/16	6.24e − 07	3.59e − 07
MF	GO:0,031,406	Carboxylic acid binding	5/16	3.29e − 05	9.06e − 06
MF	GO:0,043,177	Organic acid binding	5/16	3.29e − 05	9.06e − 06
MF	GO:0,003,713	Transcription coactivator activity	5/16	1.72e − 04	4.75e − 05
MF	GO:0,005,504	Fatty acid binding	3/16	3.57e − 06	1.12e − 04
MF	GO:0,033,293	Monocarboxylic acid binding	4/16	3.29e − 05	9.06e − 06

**Table 2:** Results of KEGG enrichment analysis.

Ontology	ID	Description	GeneRatio	*p*.value	*p*.adjust
KEGG	hsa00592	Alpha-Linolenic acid metabolism	2/15	9.42e − 04	0.006
KEGG	hsa00410	Beta-Alanine metabolism	2/15	0.001	0.008
KEGG	hsa01040	Biosynthesis of unsaturated fatty acids	3/15	1.48e − 05	2.04e − 04
KEGG	hsa00071	Fatty acid degradation	3/15	6.56e − 05	6.05e − 04
KEGG	hsa05215	Prostate cancer	3/15	6.88e − 04	0.006
KEGG	hsa04922	Glucagon signaling pathway	3/15	9.16e − 04	0.006
KEGG	hsa04931	Insulin resistance	3/15	9.41e − 04	0.006
KEGG	hsa04068	FoxO signaling pathway	3/15	0.002	0.009
KEGG	hsa01212	Fatty acid metabolism	4/15	2.87e − 06	5.96e − 05
KEGG	hsa04920	Adipocytokine signaling pathway	4/15	6.21e − 06	1.03e − 04
KEGG	hsa04919	Thyroid hormone signaling pathway	4/15	5.76e − 05	5.98e − 04
KEGG	hsa04146	Peroxisome	5/15	2.81e − 07	1.17e − 05
KEGG	hsa01200	Carbon metabolism	5/15	1.63e − 06	4.52e − 05
KEGG	hsa04024	cAMP signaling pathway	5/15	3.15e − 05	3.74e − 04
KEGG	hsa03320	PPAR signaling pathway	7/15	3.60e − 11	2.99e − 09

### Results of GSEA analysis of genes associated with diabetic peripheral neuropathy

**Figure 4:**
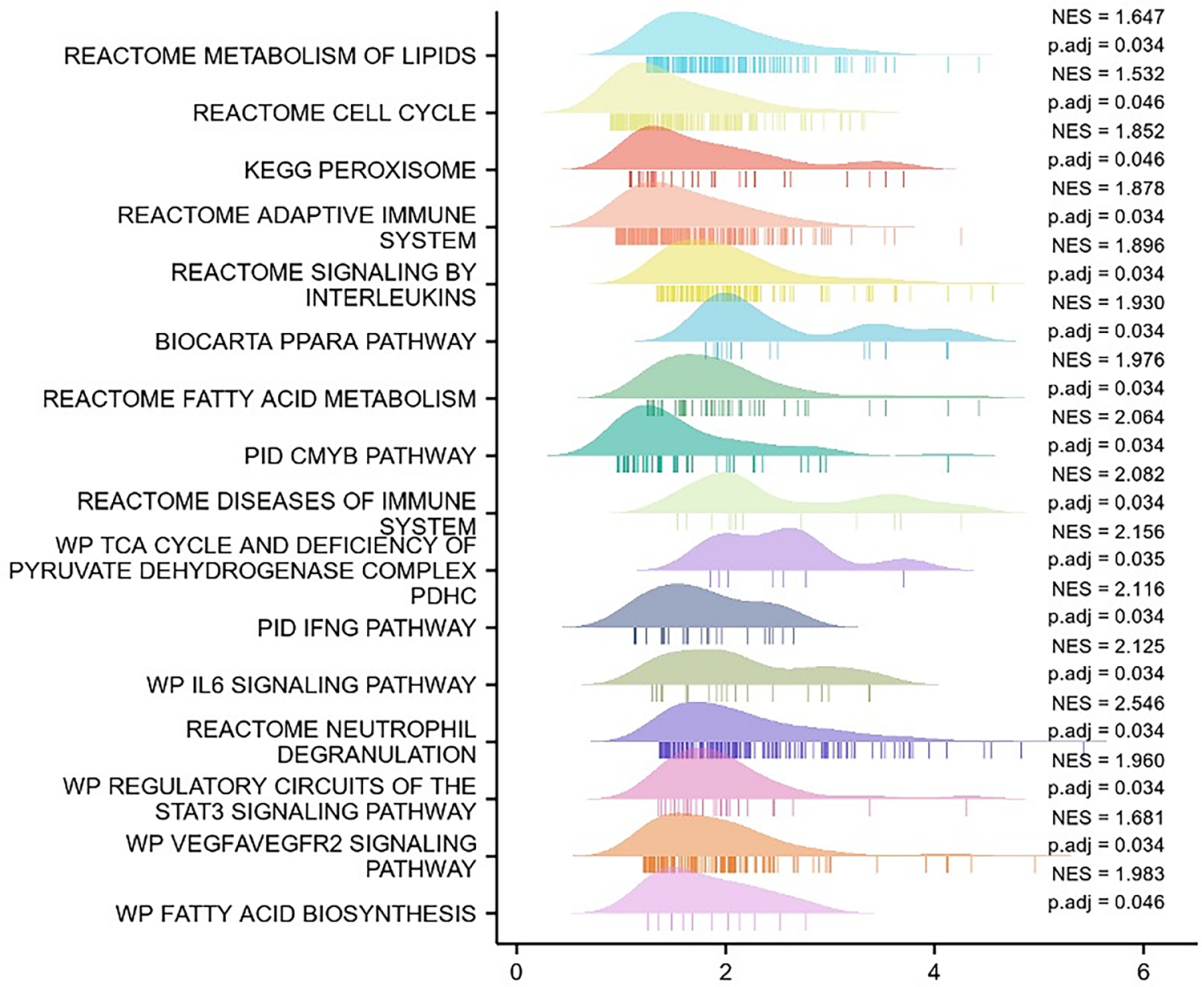
Mountain range map top16 results.

**Figure 5:**
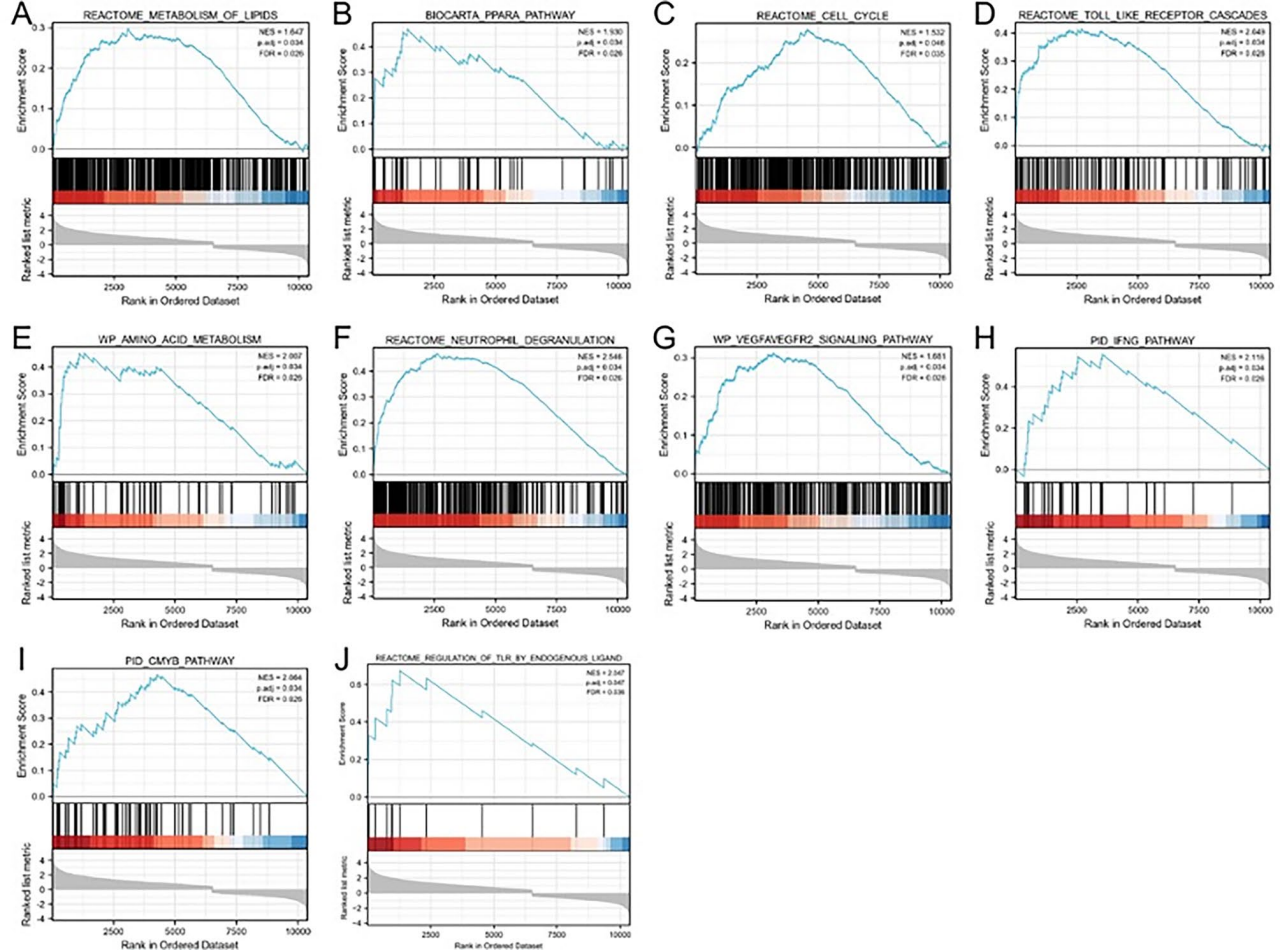
GSEA enrichment analysis. signalling pathways and biological processes that are dominant in CREBBP, EP300, ME1, CD36 in DPN and healthy samples.

To predict the function of the co-regulated DEGs and the 20 target genes, we analysed the genes in their expression profiles at an overall level using the Molecular Signatures Database through GSEA software. Compared with genes of normal human participants, a total of 16,405 genes were regulated, which were enriched using the clusterProfiler R package to analyse and explore potential functional pathways^38^. 111 gene sets were significantly enriched at a False discovery rate (FDR) < 0.25, p.adjusted < 0.05 and among the top20 pivotal genes, while PGM1, ALDH6A1, DBT, HELZ2 were not significantly enriched. The final screening of 16 pivotal genes that play a key role in the enrichment pathway associated with the onset and progression of diabetic peripheral neuropathy: ACOX1, RXRA, CREBBP, PPARA, EP300, NCOA2, ME1, HSD17B4, ACSL1, CHD9, CD36, ACOX3, HMGCL, DLAT, PTEN, and TALDO1. GSEA results showed that the most enriched biological processes were mainly the metabolic processes of linolenic acid, fatty acid oxidation using acyl coenzyme a oxidase, monocarboxylic acid transport, coenzyme metabolic processes, regulation of lipid metabolic processes, and peroxisomal substrates (Fig. 4); CREBBP, EP300, ME1, and CD34 were also specifically involved in REACTOME_ METABOLISM_OF_LIPIDS, REACTOME_NEUTROPHIL_DEGRANULATION, WP_VEGFA/VEGFR2_SIGNALING_PATHWAY, the REACTOME_TOLL_LIKE_RECEPTOR_CASCADES, PID_CMYB_PATHWAY, BIOCARTA_PPARA_PATHWAY , REACTOME_CELL_CYCLE, REACTOME_REGULATION_OF_TLR_BY_ENDOGENOUS_LIGAND and other biological processes (Fig. 5 ).

### Expression profile and screening identification of target genes associated with diabetic peripheral neuropathy

**Figure 6:**
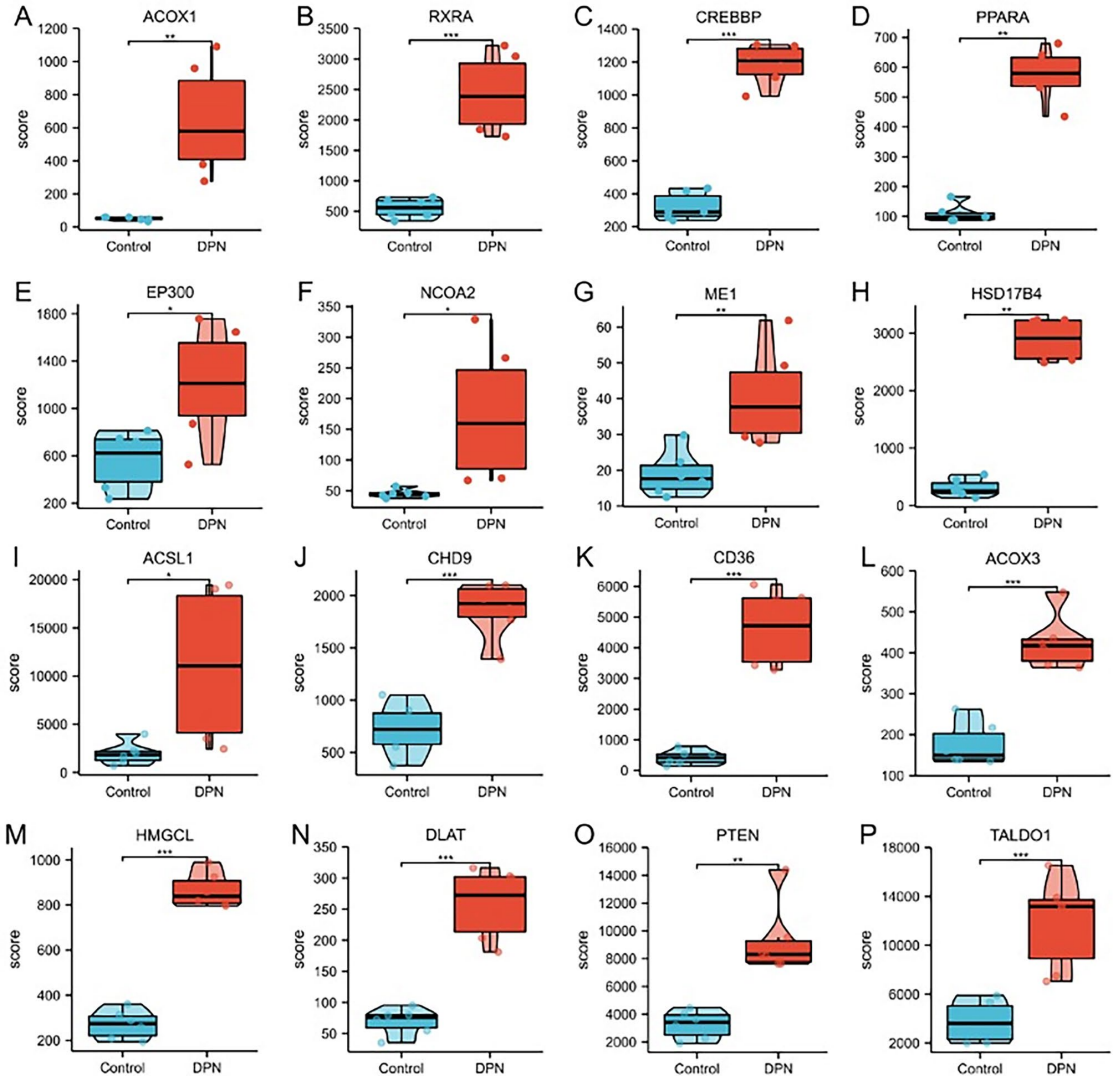
Expression of 16 hub genes in DPN versus healthy samples Comparison of the profiles of ACOX1 (**A**), RXRA (**B**), CREBBP (**C**), PPARA (**D**), EP300 (**E**), NCOA2 (**F**), ME1 (**G**), HSD17B4 (**H**), ACSL1 (**I**), CHD9 (**J**, CD36 (**K**), ACOX3 (**L**), HMGCL(**M**), DLAT(**N**), PTEN(**O**) and TALDO1 (**P**). (*p < 0.05; **p < 0.01, ***p < 0.001).

**Figure 7:**
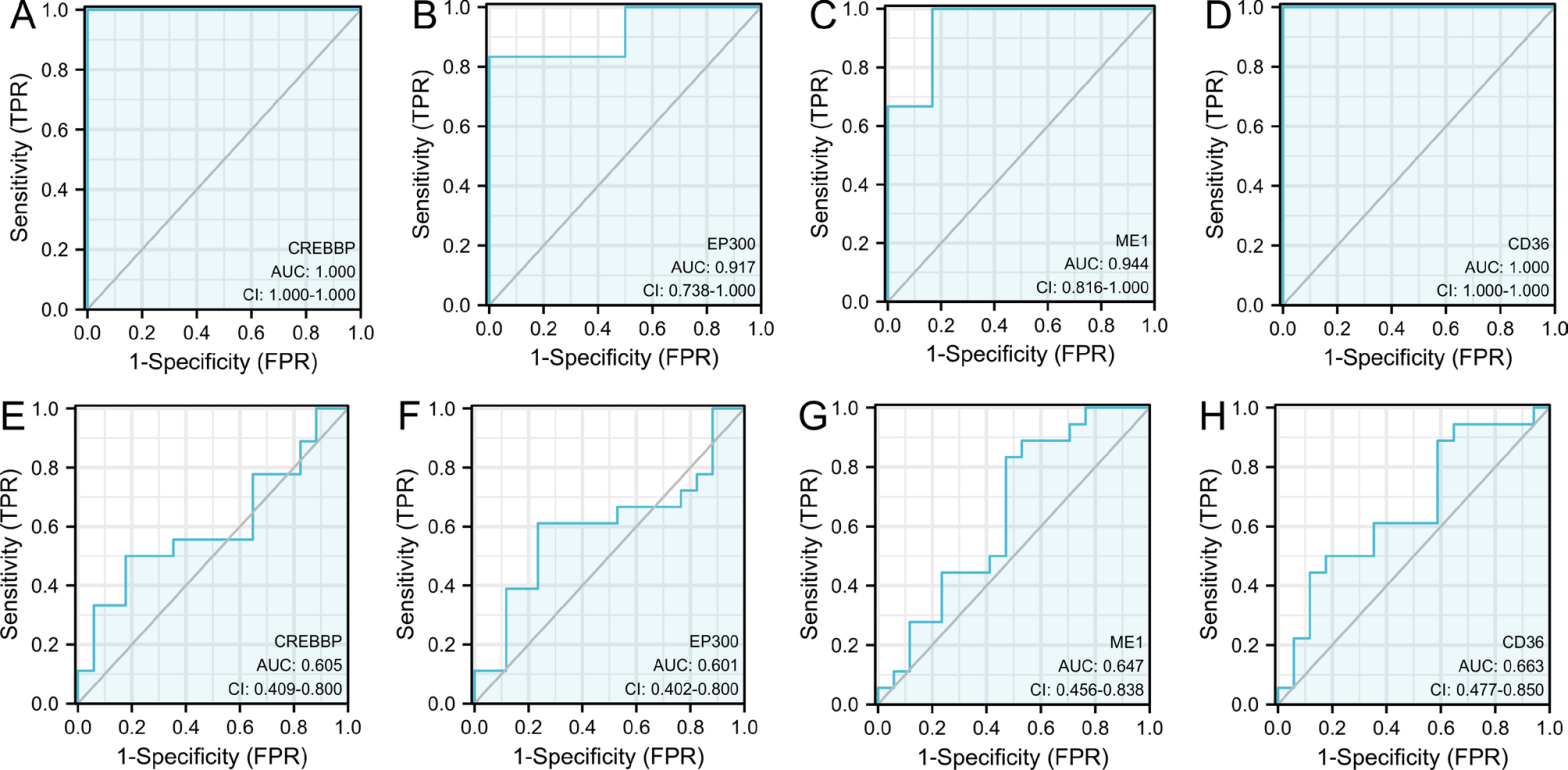
ROC diagnostic curves for four hub genes in DPN and healthy samples. (**A**–**D**) ROC curves for GSE95849. (**E**–**H**) ROC curves for GSE24290. Note: The 4 hub genes (CREBBP, EP300, ME1, CD36) are all genes with an AUC > 0.6000. Abbreviations: AUC: area under the curve; TPR: true positive rate; FPR: false positive rate.

The results of detecting the expression of the screened target genes using GSE95849 showed that the expression of 16 diabetic peripheral neuropathy-related pivotal genes (ACOX1, RXRA, CREBBP, PPARA, EP300, NCOA2, ME1, HSD17B4, ACSL1 , CHD9, CD36, ACOX3, HMGCL, DLAT PTEN, TALDO1) were consistent with the predicted expression of genes between healthy individuals, and the DPN group were higher than the Control group and the differences were statistically significant (Fig. 6). The GSE95849 was used to screen and identify target genes. To further assess the diagnostic value of target genes in diabetic peripheral neuropathy, ROC curves were established with area under the ROC curve values between 0.5 and 1. The closer the AUC was to 1, the better the diagnosis. AUC between 0.5 and 0.7 had low accuracy, AUC between 0.7 and 0.9 had some accuracy. AUC above 0.9 has a high accuracy. In the GSE95849 dataset, the variable CREBBP had high accuracy in predicting outcome in normal patients and patients with diabetic peripheral neuropathy (AUC = 1.000, CI = 1.000–1.000); the variable EP300 (AUC = 0.917, CI = 0.738–1.000); the variable ME1 (AUC = 0.944, CI = 0.816–1.000); and variable CD36 (AUC = 1.000, CI = 1.000–1.000) all had high accuracy in predictive power (Fig. 7 A-D). We also used the GSE24290 database for validation, which included 18 patients with progressive diabetic peripheral neuropathy and 17 patients with non-progressive diabetic peripheral neuropathy, to create validation ROC curves. The results showed that GSE24290 confirmed CREBBP, EP300, ME1 and CD36 were equally important diagnostically. The predictive power of the variables CREBBP (AUC = 0.605, CI = 0.409–0.800); EP300 (AUC = 0.601, CI = 0.402–0.800); ME1 (AUC = 0.647, CI = 0.456–0.838); CD36 (AUC = 0.663, CI = 0.477–0.850), was consistent with the predictions in GSE95849 (Fig. 7 E–H). Subsequently, in combination with the results of KEGG enrichment analysis, among the three key genes with high diagnostic value, CREBBP and EP300 were mainly involved in the Glucagon signaling pathway, HIF-1 signaling pathway, and Thyroid hormone signaling pathway. CD36 is mainly involved in insulin resistance, AMPK signaling pathway, Adipocytokine signaling pathway and fat digestion and absorption. (Fig. 3F, G).

### Results of network construction for mRNA and miRNA

To further evaluate the potential of circulating miRNAs as markers of diabetic peripheral neuropathy to screen for important miRNAs and mRNAs, we used the miRWalk database to predict 16 Hub genes (ACOX1, RXRA, CREBBP, PPARA, EP300, NCOA2, ME1, HSD17B4, ACSL1 , CHD9 CD36, ACOX3, HMGCL, DLAT, PTEN, TALDO1) for target miRNAs. Meanwhile, in combination with the miRDB database, 200 target miRNAs in four specifically expressed target genes were obtained by screening and 400 mRNA-miRNA pairs were determined. Based on the predicted results, a visual mRNA-miRNA network consisting of 68 nodes and 90 edges was constructed by Cytoscape . There were 42 miRNAs regulating CREBBP, 10 miRNAs regulating EP300,6 miRNAs regulating SRXN1 and 32 miRNAs regulating CD36.

**Figure 8:**
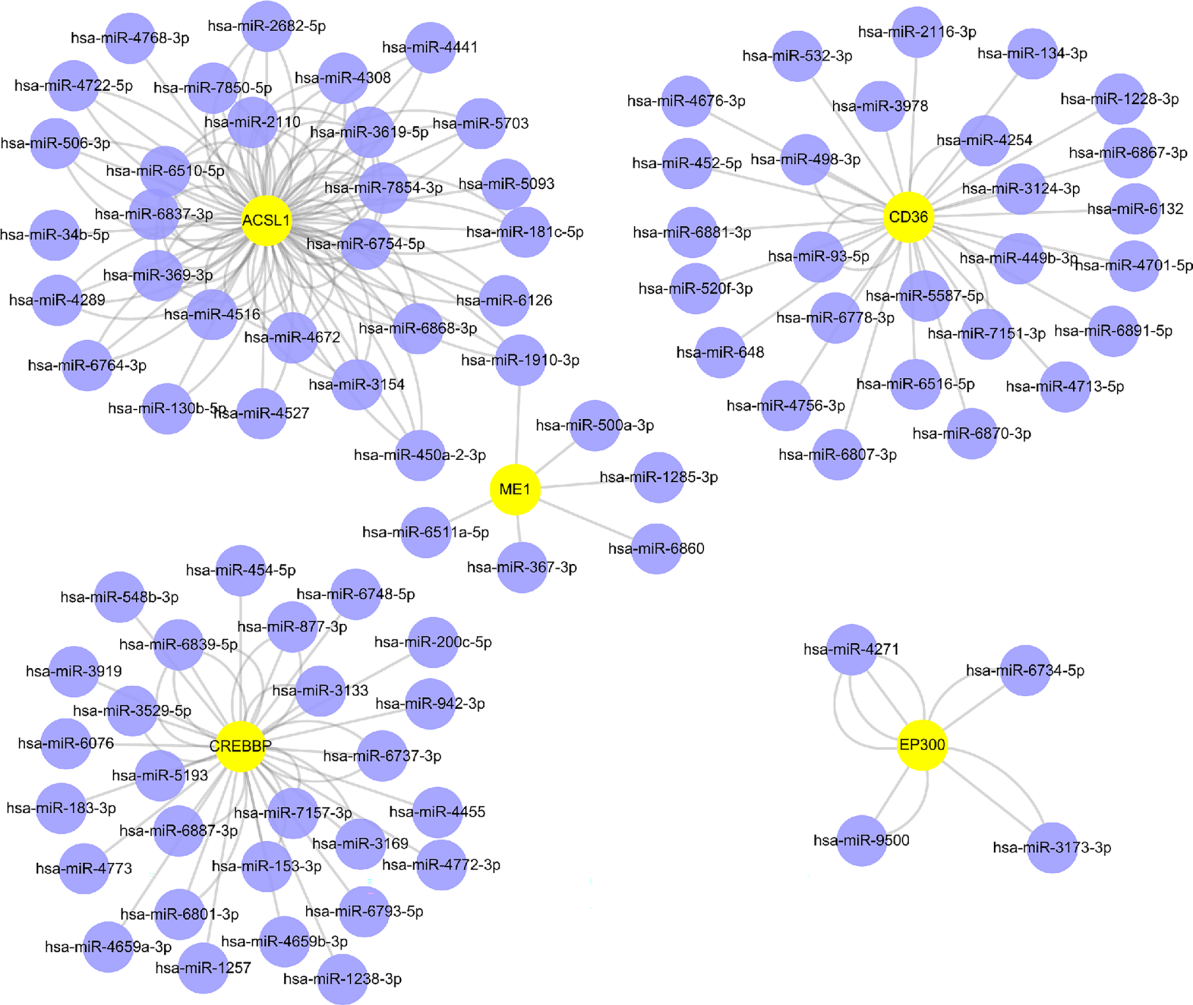
Construction of the mRNA-miRNA regulatory network of Hub genes. Construction of the mRNA (yellow)-miRNA (purple) network.

We finally used the miRTarBase database for overlay validation, which is a database that integrates microRNA targets that have been experimentally validated. Three mRNAs were obtained: CREBBP, EP300 and CD36, corresponding to three miRNAs: hsa-miR-5193, hsa-miR-3173-3p and hsa-miR-7151-3p (Fig. 8).

## Discussion

In recent years, there has been an increasing amount of research into the diagnosis and treatment of diabetic peripheral neuropathy (DPN), but due to limited understanding of the pathogenesis of DPN and the lack of specific investigational drugs, patients with DPN have a rapidly progressive disease and still have a poor prognosis. There is now some understanding of the pathogenesis of DPN and studies have shown that lipid metabolism, glucose metabolism, β-cell dysfunction, insulin resistance, mitochondrial damage, microvascular damage, microcirculatory disorders and ischaemia and hypoxia play an important role in the development and progression of DPN. However, a multifactorial aetiology involving metabolic and vascular factors remains controversial. In addition to glucose metabolism, other components of the metabolic syndrome may also play a role in the onset and progression of DPN. Of these, dyslipidemia is most closely associated with diabetic neuropathy.

In this study, we screened 353 co-expressed DEGs by analyzing the GSE95849 microarray dataset of diabetic peripheral neuropathy in the GEO database, constructed a PPI network, screened 20 hub genes, combined with GSEA, GO/KEGG enrichment analysis and correlation analysis, and performed joint validation using the GSE24290 dataset. Four target genes, namely CREBBP, EP300, ME1 and CD36, were finally obtained. 200 target miRNAs and 400 mRNA-miRNA pairs were then screened by miRWalk for miRNAs associated with the four target genes, which were experimentally validated through miRTarBase database and miRDB database. The final 3 mRNAs were obtained after database overlap of miRNA target genes: CREBBP, EP300, CD36, corresponding to 3 miRNAs: hsa-miR-5193, hsa-miR-3173-3p, hsa-miR-7151-3p. In this study we found that the enriched GO classes were macrophage-derived processes such as foam cell differentiation, regulation of lipid metabolism, reduced oxygen content, positive regulation of the TGF-β receptor signalling pathway, positive regulation of Notch receptor target transcription, cellular carbohydrate metabolism, glucose metabolism, response to hypoxia, lipid transport, fatty acid metabolism, and coenzyme metabolism. In the KEGG enrichment analysis, DEGs were mainly involved in metabolic processes such as PPAR signalling pathway, CAMP signalling pathway, insulin resistance, and carbon metabolism. Among them, TGF-β is a pleiotropic cytokine, which is involved in inflammatory response. After neurological injury, TGF-β regulates the behaviour of neurons and glial cells, thus mediating the regenerative process^49^. Therefore, the upregulation of TGF-β signalling may be a pathological response to neurological injury. Lipoic acid is a common antioxidant for the treatment of DPN, while TGF-β increases lipid peroxidation and lipoic acid decreases TGF-β expression^50^. Lipoic acid reduces TGF-β expression.

In our study, we combined KEGGF enrichment analysis and GSEA analysis to identify potential biomarkers and biological pathways in DPN, which ultimately yielded different results. Through screening of key genes and reviewing the literature, this study suggests that CREBBP, EP300, CD36 may be key genes for DPN occurrence. The results of this study show that CREBBP, EP300, CD36 are also important for the diagnosis of DPN, the pathogenesis of diabetic peripheral neuropathy is caused by the interaction of multiple pathways, and the pathogenesis is complex, requiring a comprehensive multi-target-multi-pathway analysis.

P300 (EP300) and CBP (CREBBP) have at least 315 different cellular and viral interacting proteins and are considered to be the most tightly connected coactivators in the mammalian protein–protein interaction network^51,52^. Although there are two separate genes encoding CBP and p300, they share 61% sequence homology and are often referred to as p300/CBP^53^. p300 and CBP are transcriptional co-activators with histone acetyltransferase activity. A variety of B-cell transcription factors can recruit p300/CBP, and thus coactivators are important for B-cell function and health. It has been shown that competition for cellular transcription factors because of binding of restricted p300/CBP is an important regulator of transcription. So when this micro-competitive regulation is disrupted, it causes many diseases^54^. It is well documented that p300/CBP affects lipid metabolism in different tissues and cells. Furthermore, in mouse models of obesity and type 2 diabetes, high p300/CBP HAT activity is associated with ChREBP hyperacetylation and hepatic steatosis^55^. In our study, p300(EP300) and CBP(CREBBP) expression levels were upregulated in DNP patients, but no studies on p300(EP300), CBP(CREBBP) and DPN have been identified. The present study is the first to focus on the role of p300(EP300) and CBP(CREBBP) in DPN, and we would like to know whether p300(EP300) and CBP(CREBBP) affect DPN in DPN by influencing lipid metabolism toxicity, or by inflammatory processes induced by inflammatory stimuli. In any case, the exact mechanisms need to be further investigated.No studies have been conducted on P300 (EP300) and CBP (CREBBP) in the context of DPN. Relevant study on P300 (EP300) and CBP (CREBBP) and diabetic complications is that EP300 also play pro-apoptotic roles in neuron^56,57^.

CD36 is a key mediator of ox-LDL uptake by macrophages and has received much attention. CD36 regulates a variety of physiological and pathological processes, including FA transport and lipid metabolism, angiogenesis, adhesion, inflammation, cardiomyopathy, diabetes and atherosclerosis. CD36 independently binds and recognises a variety of exogenous or endogenous ligands, including those found in pathogenic or pathogen-infected cells, apoptotic cells, long-chain fatty acids (LCFA), modified low-density lipoproteins (LDL) and high-density lipoproteins (HDL)^58^. Patients with CD36 deficiency or CD36 gene polymorphism often present with postprandial hyperlipidaemia and high levels of plasma apoB48, triglycerides, FA and celiac (CM) residues^59,60^. These observations suggest a role for CD36 in hyperlipidemia. These observations suggest the importance of CD36 in hyperlipidaemia and associated atherosclerosis. CD36 is involved in multiple processes of lipid metabolism, including dietary lipid intake, lipoprotein production and transport, lipid utilisation, storage and lipolysis, which is consistent with our sample selection and grouping and bioinformatic predictions. It’s worth noticing that CD36 has been reported in several metabolic diseases, but there is currently no literature suggesting a relationship between CD36 and DPN. Our study suggests that this may be a novel prognostic factor in diabetic peripheral neuropathy and further studies are needed to investigate the mechanism of its role in DPN.CD36 facilitates the transport of free fatty acids across the cell membrane in adipocytes. Downregulation of CD36 in progressive DPN may reduce lipid uptake and affect myelin formation, but may exert a protective effect^61^.

CD36 is implicated in the initiation of Peripheral nerve inflammation. The upregulations.of CD36 and MAPK signaling pathway genes (TNF-α, IL-1a and TGF-β1) are closely associated with the nerves of BKS db/db mice and the some studies suggest a CD36-mediated inflammatory response^62^. Moreover, it is suspected that CD36 also modulates energy homeostasis-related signaling pathways, such as AMPK and PPAR pathways, changing the glucose and lipid metabolism in diabetic peripheral nerves^63^. Dyslipidaemia was shown to predispose to the dysregulation of lipid metabolism in peripheral nerves, correlated with upregulation of CD36 and diacylglycerol acyltransferase 2. Saturated FAs are incorporated into TAGs, which initiate nerve injury ^64^.

At the same time, due to the widespread use of bioinformatics for gene chip analysis methods, we offer reasonable speculation on the reasons for the inconsistent expression levels of mi-RNAs in the development of diabetic peripheral neuropathy. The specific mechanisms of mi-RNA interactions with cytokines and their involvement in signalling pathways in disease progression require more exploration and experimentation. Similarly, the microRNAs identified in our current study, miRNA5193, miRNA3173 and miRNA7151, are associated with disease progression in diabetic peripheral neuropathy, but their specific targets of action in clinical disease development and related clues still need further investigation. The current study found that miRNA5193 down-regulates TR1M11 expression in prostate cancer^65^.miR-5193 is an essential suppressor of ovarian cancer development and an important downstream regulator of FUT1 carcinogenesis in ovarian cancer^66^. In the future, miR-5193 may play an important role in the inhibition of HBV replication^67^. Meanwhile, studies have shown that^68^ downregulation of SNHG3 expression suppressed the malignant phenotype of cholangiocarcinoma cells through the miR-3173-5p/ERG axis^69^ . Recent studies have shown that^70^ CASC15 can act as an endogenous miRNA sponge to uptake and downregulate miR-7151-5p, thereby preventing the inhibition of WNT7A during papillary thyroid cancer progression.

Our study has some limitations. Firstly, this study was based on a bioinformatics analysis of transcriptome profiles from public databases, which may differ from the actual situation. Only one dataset was used for screening and experimental validation of some miRNA-mRNA pairs was lacking. Secondly, although the four genes screened have previously been reported to mediate diabetes and metabolism-related diseases, there is no direct evidence that they regulate the onset, progression and prognosis of diabetic peripheral neuropathy. Therefore, further experimental evidence is needed to validate the specific regulatory functions of these genes in diabetic peripheral neuropathy. Finally, prospective clinical trial cohorts and more in-depth molecular biology experiments need to be designed and conducted to further validate the mechanism of action of these four related genes in the development of diabetic peripheral neuropathy.

The necessity and clinical significance of this study lies in that, given the current state of medical development, there is no cure for diabetes, thus it is particularly important to actively prevent diabetes complications in people who have not yet developed them, to improve DM patients’ lifestyle, to improve their quality of living and to reduce disability and mortality. Finding the right target for treatment, giving individualised treatment plans and more targeted treatment can reduce the economic pressure on patients, families and even the society as a whole.

In summary, the candidate genes CREBBP, EP300, CD36, miRNA5193, miRNA3173 and miRNA7151, which were screened based on bioinformatics analysis, can influence the process of diabetic peripheral neuropathy through lipid metabolism, TGF-β receptor signaling pathway, lipid transport and PPAR signaling pathway. They may play an important role in the clinical disease progression of diabetic peripheral neuropathy, providing meaningful research clues and directions for clinical prognosis determination and treatment.

## Data Availability

The sequencing data used to support the findings of this study have been deposited in the GEO repository (GSE95849 and GSE24290). The datasets generated and/or analysed during the current study are available in the NCBI repository, https://www.ncbi.nlm.nih.gov/geo/query/acc.cgi?acc=GSE95849, https://www.ncbi.nlm.nih.gov/geo/query/acc.cgi?acc=GSE24290.
